# Editorial: Imaging elasticity as a tangible prognostic factor in cancers

**DOI:** 10.3389/fonc.2023.1211615

**Published:** 2023-06-21

**Authors:** Richard G. Barr

**Affiliations:** Radiology, Northeastern Ohio Medical University, Youngstown, OH, United States

**Keywords:** elastography, cancer, breast, tumor response, shear wave

In this Research Topic area of using elastography as a prognostic factor in cancers, five articles have been published. To show the robustness of the technique, these articles evaluate breast cancer, lymph node cancer, carotid body tumors, and colon cancer. A review of MRE for tumor diagnosis is also included in this topic area.

Most cancers are stiff. When treatments are working, the cancers become soft. This allows elastography to be an excellent biomarker for treatment response. Further work is needed to determine the amount of change in elasticity needed to confirm that a treatment is working over a short period of time. If that early response is not seen, the patient can be changed to another treatment regimen. The changes in elastography can predict a complete response, which also needs to be determined. These changes will probably be different for each type of cancer. Investigation of whether the percentage of the tumor that is changing stiffness early on is indicative of treatment response, size changes, or other additional features will improve the accuracy of treatment response. It is also important to note that some cancers such as lymphoma are not stiff, and it is unlikely that elastography will be able to predict treatment response in these patients.

Most other imaging for early treatment response requires injection of a contrast agent, and it is change in vascularity that is used to assess treatment response. The RECIST criteria have been used, but there is a delay in finding the changes that occur, and therefore, early response to treatment is usually not observed. Elastography is performed without contrast and therefore can be used in patients who cannot receive contrast. Also, ultrasound elastography is less expensive and can be performed at more time points if needed.

Further work in this area is needed to develop protocols for the use of elastography for treatment monitoring. Multicenter trials using these protocols are also needed for elastography to become an accepted method of treatment monitoring.

## Author contributions

The author confirms being the sole contributor of this work and has approved it for publication.

